# Differences in Redox Regulatory Systems in Human Lung and Liver Tumors Suggest Different Avenues for Therapy

**DOI:** 10.3390/cancers7040889

**Published:** 2015-11-10

**Authors:** Ryuta Tobe, Bradley A. Carlson, Petra A. Tsuji, Byeong Jae Lee, Vadim N. Gladyshev, Dolph L. Hatfield

**Affiliations:** 1 Molecular Biology of Selenium Section, Mouse Cancer Genetics Program, National Cancer Institute, National Institutes of Health, Bethesda, MD 20892, USA; carlsonb@mail.nih.gov; 2 Department of Biological Sciences, Towson University, Towson, MD 21252, USA; ptsuji@towson.edu; 3 School of Biological Sciences, Seoul National University, Seoul 151-742, Korea; imbglmg@snu.ac.kr; 4 Department of Medicine, Brigham & Women’s Hospital, Harvard Medical School, Boston, MA 02115, USA; vgladyshev@rics.bwh.harvard.edu

**Keywords:** antioxidant systems, thioredoxin reductase 1, thioredoxin 1, glutathione, glutathione peroxidase

## Abstract

A common characteristic of many cancer cells is that they suffer from oxidative stress. They, therefore, require effective redox regulatory systems to combat the higher levels of reactive oxygen species that accompany accelerated growth compared to the normal cells of origin. An elevated dependence on these systems in cancers suggests that targeting these systems may provide an avenue for retarding the malignancy process. Herein, we examined the redox regulatory systems in human liver and lung cancers by comparing human lung adenocarcinoma and liver carcinoma to their respective surrounding normal tissues. Significant differences were found in the two major redox systems, the thioredoxin and glutathione systems. Thioredoxin reductase 1 levels were elevated in both malignancies, but thioredoxin was highly upregulated in lung tumor and only slightly upregulated in liver tumor, while peroxiredoxin 1 was highly elevated in lung tumor, but downregulated in liver tumor. There were also major differences within the glutathione system between the malignancies and their normal tissues. The data suggest a greater dependence of liver on either the thioredoxin or glutathione system to drive the malignancy, while lung cancer appeared to depend primarily on the thioredoxin system.

## 1. Introduction

Cancer cells are characterized by hallmarks that set them apart from normal cells, and one of these hallmarks is uncontrolled cell growth [1]. Cancer cells must depend on strong antioxidant systems to combat the burdens of oxidative stress generated by elevated levels of reactive oxygen species (ROS) accompanying unrestrained malignant growth and serving as both “Savior and Satan” to cell augmentation (e.g., see [2,3,4,5,6,7,8,9] and references therein). Two of the major redox regulatory systems in mammals that support increased tumor growth are the thioredoxin (TXN) and the glutathione (GSH) systems [10,11,12,13,14,15,16,17,18,19,20]. The principal functions of these systems are to maintain redox homeostasis at the level of protein-based and low molecular weight thiols, and both systems are comprised of several selenium-containing enzymes (selenoproteins) whose expression levels are influenced by levels of selenium in the diet. Cancer cells, however, often become increasingly dependent on these systems as well as on other antioxidants in dealing with excessive oxidative damage [10,11,12,13,14,15,16,17,18,19,20]. In recent years, one of the primary foci regarding the forces driving cancer growth has been the disrupted redox homeostasis that accompanies rapid growth. Most certainly, antioxidants used by cancer cells vary among different cancers suggesting different avenues of therapy [10,11,12,13,14,15,16,17,18,19,20].

The available evidence suggests that lung adenocarcinoma (non-small cell) and hepatocellular carcinoma and the corresponding cancer cells utilize antioxidant systems differently to sustain their malignancies [10,11,12,16,21,22,23]. Furthermore, in liver cancer, the incidence of malignancy in mice maintained on a selenium adequate diet and carrying liver cancer driver transgenes (TGFα/c-myc) [24], or in mice exposed to the liver carcinogen, diethylnitrosamine (DEN) [25], was enhanced compared to mice maintained on a selenium deficient or a highly enriched selenium diet. The latter studies suggested that mouse diets which are selenium deficient or supplemented with high levels of selenium (2.0 ppm selenium) provide the animals with greater protection from hepatocarcinogenesis than diets with adequate levels of selenium (0.1 ppm selenium). The role of varying selenium levels in preventing and promoting cancer has been reviewed recently providing further examples of selenium deficiency and dietary selenium supplementation in these processes (see reviews in [26,27,28,29]).

Mice lacking the selenoprotein thioredoxin reductase 1 (TXNRD1) in liver manifested a dramatically higher incidence of tumor development than control mice expressing TXNRD1 when both mouse lines were treated with a liver carcinogen, DEN [30]. Tumors from the TXNRD1-deficient mice exhibited upregulated expression of enzymes involved in the GSH system and elevated levels of another selenoprotein, glutathione peroxidase 2 (GPX2), as well as an enrichment of additional nuclear factor (erythroid-derived 2)-like 2 (NRF2)-regulated enzymes. These increases in NRF2-regulated enzymes likely compensated for the oxidative burden of TXNRD1-deficient hepatoma cells once tumor formation occurred (see also [31,32,33,34,35]). Lung epithelial cells, on the other hand, appear to utilize selenoproteins and selenium in a different manner than hepatocytes in protecting lung from oxidative stress and disease. The targeted removal of TXNRD1 in a mouse lung cancer cell line, LLC1 cells, resulted in the cells reversing many of their malignant properties to those more similar to normal cells, including a dramatic reduction in tumorigenic and metastatic properties [36]. The data suggested that the reduction in TXNRD1 levels in lung cancer cells is anti-tumorigenic. TXNRD1-deficient A549 human lung cancer cells, however, were found to have only minor changes in growth properties compared to control TXNRD1-sufficient cells, but no other apparent phenotypic alterations [37]. Even though the knockdown of TXNRD1 in these cells was almost 90%, the authors concluded that sufficient amounts of this enzyme remained to keep TXN in the reduced state. Interestingly, A549 TXNRD1-deficient cells were highly sensitive to the drugs, 1-chloro-2,4-dinitrobenzene and menadione, but not to auranofin and juglone, compared to control cells, suggesting roles of this selenoenzyme independent of TXN [37].

Furthermore, the serum selenium levels were examined in 3333 males over a 16 year period to assess whether low selenium levels were associated with increased lung cancer incidence [38]. Although low levels of serum selenium levels were not found to be involved with increased lung cancer risk, a significantly greater risk from lung cancer mortality was observed among heavy smokers with high serum selenium levels suggesting that higher selenium levels may drive malignancy. Other studies have reported that low selenium levels may be associated with lung cancer [39,40], while one of these studies cautioned against the use of selenium as a strategy to prevent lung cancer [39]. A human clinical trial involving the recurrence of non-small cell lung cancer in patients given a placebo or selenium supplementation showed no difference between the two groups resulting in early termination of the trial [41]. Interestingly, a recent study has shown that the time required for lung tumorigenesis development was reduced and the resulting tumor size increased dramatically by adding vitamin E or N-acetylcysteine to the diet of mice carrying a lung cancer driver gene compared to untreated control mice carrying the same cancer driver gene [21].

GSH is an abundant intracellular tripeptide with many biological roles, including protection against reactive oxygen species (ROS). The GSH system consists of enzymes involved in GSH synthesis, glutamate-cysteine ligase (GCLC) and glutathione synthetase (GSS), as well as glutathione peroxidases, glutaredoxins, and glutathione *S*-transferases. Furthermore, GSH is involved in a number of vital cell processes such as maintenance of the redox state, detoxification of xenobiotics and endogenous compounds, and DNA synthesis [20,42,43,44,45], including cross-talk between the GSH and TXN systems [12,16]. Additionally, GSH levels, as well as other components of the GSH system, have been shown to be elevated in many human cancers [46].

The above studies suggest that lung and liver tumors have altered their antioxidant systems and/or proteins to support their malignancies. To elucidate the antioxidants and redox regulators used by human liver and lung cancers, we examined them in greater detail in these two cancers.

## 2. Results

### 2.1. Lung and Liver Tumor and Normal Tissues

We examined lung adenocarcinomas (Table 1) and hepatocellular carcinomas (Table 2) and the respective surrounding normal tissues from three lung cancer and three liver cancer patients. Each tissue was closely matched in terms of percent malignant cells within the tumor, tumor grade and stage, as well as patient sex and age. Patients were free of other known diseases, and normal tissues surrounding tumors were free of malignancy as determined by histopathological analysis. All lung adenocarcinomas were non-small cell carcinomas.

**Table 1:** Lung samples ^1^.

Sample No.	Name	Pathological Diagnosis ^2^	Cancer Cell %	Grade	Stage	Sex	Age
1	N1	Normal Adjacent Tissue	-	-	-	M	48
	T1	Adenocarcinoma	70%	2	IIB		
2	N2	Normal Adjacent Tissue	-	-	-	M	56
	T2	Adenocarcinoma	70%	2	IIB		
3	N3	Normal Adjacent Tissue	-	-	-	M	59
	T3	Adenocarcinoma	70%	2	IIB		

**^1^** None of the patients had hepatitis B or C, or were HIV positive and all patients were free of other diseases; ^2^ All lung adenocarcinomas were non-small cell carcinomas.

**Table 2:** Liver samples ^1^.

Sample No.	Name	Pathological Diagnosis	Cancer Cell %	Grade	Stage	Sex	Age
1	N1	Normal Adjacent Tissue	-	-	-	M	64
	T1	Hepatocellular Carcinoma	90%	2	II		
2	N2	Normal Adjacent Tissue	-	-	-	M	50
	T2	Hepatocellular Carcinoma	90%	2	II		
3	N3	Normal Adjacent Tissue	-	-	-	M	63
	T3	Hepatocellular Carcinoma	90%	2	II		

^1^ None of the patients had hepatitis B or C, or were HIV positive and all patients were free of other diseases.

### 2.2. Lung and Liver Tumors Utilize the TXN System Differently

**Figure 1:**
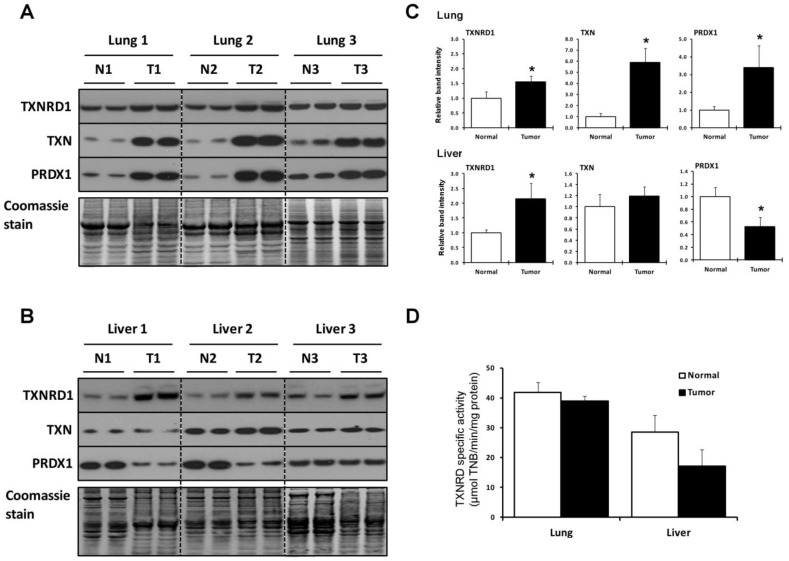
Expression of proteins of the TXN system. (**A**,**B**) Expression of TXNRD1, TXN and PRDX1 in normal and tumor lung and liver tissues, respectively, as analyzed by western blotting. N designates normal tissue and T, tumor tissue. Coomassie blue staining is shown in the bottom panels and used as a control for protein loading; (**C**) Quantification of band intensities on western blots. Relative band intensities of duplicate lanes were quantified as described in the Experimental Section and tumor sample (T) values were normalized to the control tissue (N). * Denotes statistical differences, *p* < 0.05; (**D**) TXNRD catalytic activities in normal and tumor tissues. Activities are expressed as µmol of TNB/min/mg of protein for lung (upper panel) and liver tissues (lower panel). Values are the means ± S.D. of three independent experiments. Experimental details are given in the Experimental Section.

The amounts of three key proteins in the TXN system, TXNRD1, TXN, and peroxiredoxin 1 (PRDX1), in tumor and surrounding normal tissue samples from the three lung and three liver cancer patients were examined in duplicate by western blotting (Figure 1A–C). All three proteins increased significantly in lung tumors compared to normal, surrounding tissues. The tumors from the three liver cancer patients also manifested a significantly higher increase in TXNRD1 levels (Figure 1B and lower, right panel in Figure 1C). TXN levels remained fairly constant in liver tumors compared to normal tissues, while PRDX1 levels decreased. The increases in TXNRD1 were approximately 1.5 fold in lung tumors and approximately 2 fold in liver tumors. It is not clear why liver sample 3 manifested lower levels of PRDX1 in the normal sample compared to the other two normal tissues, albeit this sample appeared to have slightly enriched amounts of this enzyme compared to the corresponding cancerous tissue. Further discussion regarding variability of analyzed proteins within normal samples and within tumor samples is given in Section 2.3.

The TXNRD enzymatic activity was also measured in lung and liver tumor and normal tissues (Figure 1D). It was approximately the same in lung tumors, but lower in liver tumors compared to normal tissues. The TXNRD activity was 25% higher in normal lung compared to normal liver tissue. The differences in the amounts of TXNRD1 protein as assessed by western blotting and TXNRD activity, and variations in the three proteins involved in the TXN system in normal and malignant tissues between the two organs are further considered below.

### 2.3. Lung and Liver Tumors Utilize the GSH System Differently

**Figure 2:**
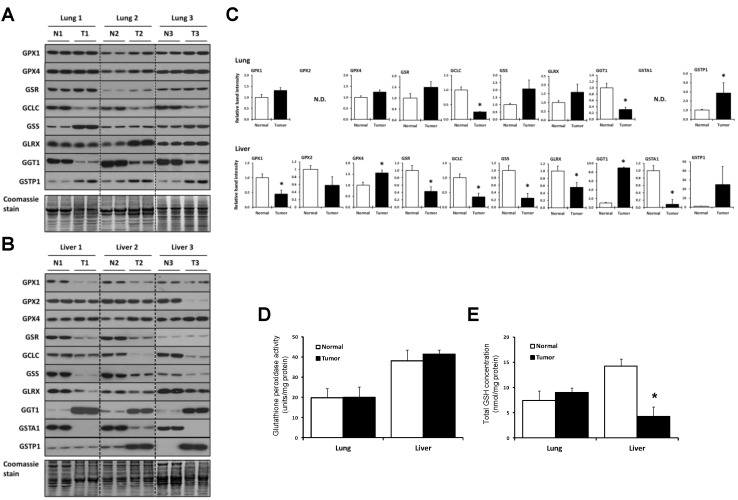
Expression of proteins of the GSH system. (**A**,**B**) Expression of GPX1, GPX2, GPX4, GSR, GCLC, GSS, GLRX, GGT1, GSTA1, and GSTP1 in normal and tumor lung and liver tissues, respectively, as analyzed by western blotting. GSTA1 and GPX2 were only detected in liver samples and therefore not included with the lung samples. N designates normal tissue and T, tumor tissue. Coomassie blue staining is shown in the bottom panels and used as a control for protein loading; (**C**) Quantification of band intensities on western blots. Relative band intensities of duplicate lanes were quantified as described in the Experimental Section and tumor sample values were normalized to the control tissue (N). * Denotes statistical differences, *p* < 0.05; (**D**) Glutathione peroxidase activities in normal and tumor tissues. Activities are expressed as units/mg of protein for lung (upper panel) and liver tissues (lower panel). Values are the means ± S.D. of three independent experiments. Experimental details are given in Experimental Section; (**E**) Amounts of total GSH (GSH + GSSG) in lung (upper panel) and liver tissues (lower panel). Total GSH levels are expressed as nmol per mg of protein and are the means ± S.D. for three independent experiments. Details are given in the Experimental Section.

The expression of enzymes involved in GSH metabolism, including glutathione reductase (GSR), GCLC, GSS, glutaredoxin (GLRX), γ-glutamyltransferase 1 (GGT1), glutathione *S*-transferase alpha 1 (GSTA1), and glutathione *S*-transferase pi 1 (GSTP1), as well as three selenoproteins, glutathione peroxidase 1 (GPX1), GPX2, and glutathione peroxidase 4 (GPX4), in tumor and surrounding normal tissue samples from the three lung and three liver cancer patients were examined in duplicate by western blotting (Figure 2A–C). The levels of GPX1, GPX4, GSR, GSS, and GLRX appeared to increase, but not significantly, in the three lung tumor samples (Figure 2A and upper panels of Figure 2C), although GSS in patient 1 and GLRX in patient 2 appeared to increase more dramatically on western blots than did the corresponding proteins in the other patients. GSTP1 increased significantly in lung tumor, while GCLC and GGT1 decreased significantly in lung tumor compared to normal tissue. GSTA1 and GPX2 were not detected in normal lung tissue or the corresponding malignant tissue.

Protein concentrations of six of the GSH system enzymes, GPX1, GSR, GCLC, GSS, GLRX, and GSTA1, decreased significantly in liver tumors (Figure 2B and lower panels in Figure 2C). GPX4 increased in tumors, while GGT1 manifested the most dramatic increase. GSTP1 increased dramatically in two of the liver tumors, but was unaffected in one of the liver samples. The levels of GPX2 and GCLC appeared to decrease in tumors, but not significantly.

**Figure 3:**
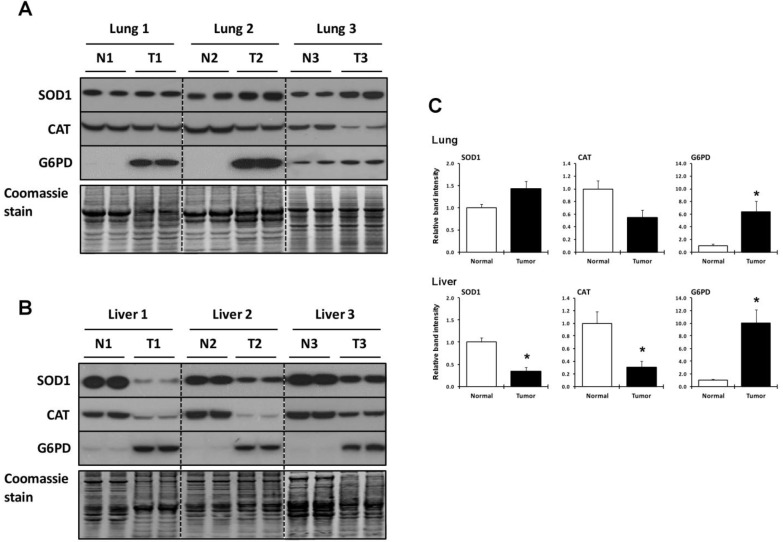
Expression of SOD1, CAT and G6PD. (**A**,**B**) Expression of SOD1, CAT and G6PD in normal and tumor lung and liver tissues as analyzed by western blotting. N designates normal tissue and T, tumor tissue. Coomassie blue staining is shown in the bottom panels and used as a control for protein loading; (**C**) Quantification of band intensities on western blots. Relative band intensities of duplicate lanes were quantified as described in the Experimental Section and tumor sample (T) values were normalized to the control tissue (N). * Denotes statistical differences, *p* < 0.05.

While variability among liver samples within normal and within malignant tissues (e.g., GPX2, GSR, GSS, and GSTP1) and among lung samples within normal and within malignant tissues (GSS, GLRX (Figure 2) and G6PD (Figure 3) below) may be attributed to the low number of sample sizes, we raise the possibility that each of the tumors may also be in slightly different transition stages in developing their dependency on specific enzymes to support the cancer.

The GPX activity and the GSH levels were also measured (Figure 2D,E, respectively). The GPX activity in the liver was about twice that in the lung, but its activity in tumors compared to normal tissues was similar (Figure 2D). The levels of GSH in lung tumor and the corresponding normal tissue were similar, but normal liver tissue had about twice the amount of GSH than lung; and the level of GSH in liver tumor was reduced significantly compared to normal tissue (Figure 2E).

### 2.4. Utilization of Other Antioxidant Proteins and Compounds in Lung and Liver Tumor and Normal Tissues

Expression of additional antioxidant proteins, superoxide dismutase 1 (SOD1), catalase (CAT), and glucose-6-phosphate dehydrogenase (G6PD), were also examined in lung and liver tumor and normal tissues by western blotting (Figure 3). SOD1 appeared to increase slightly and CAT to decrease, but not significantly, in lung tumor, while G6PD increased several fold (Figure 3A and upper panels of Figure 3C). Liver tumor tissues manifested significant decreases in SOD1 and CAT and, like lung, a highly significant increase in G6PD (Figure 3A and lower panels of Figure 3C).

**Figure 4:**
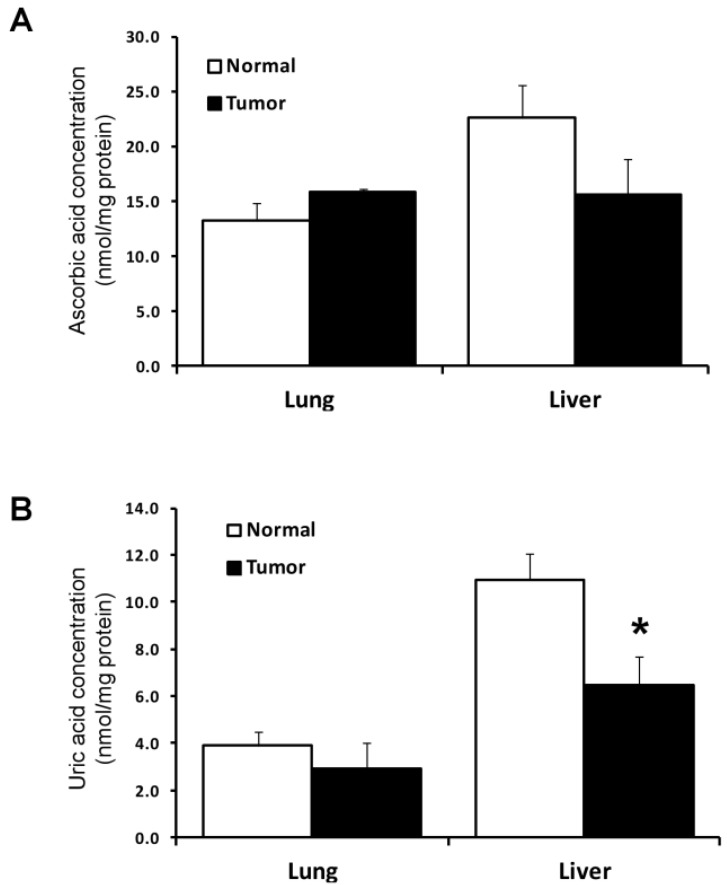
Concentrations of ascorbic acid and uric acid. (**A**,**B**) Amounts of ascorbic acid and uric acid, respectively, in lung (upper panel) and liver tissues (lower panel). The concentration of ascorbic acid and uric acid are expressed as nmol per mg of protein and are the means ± S.D. for three independent experiments. * Denotes statistical difference, *p* < 0.05. Experimental details are given in the Experimental Section.

The levels of two compounds which are considered to be antioxidants, ascorbic acid and uric acid, were also measured in the tumor and normal tissues (Figure 4). In normal liver tissues, the amounts of the compounds were about twice those in normal lung tissues. The amounts of these two antioxidants in each normal and tumor tissue were similar with the exception of uric acid in liver which was significantly lower in tumor than normal liver tissue.

## 3. Discussion

TXNRD1 is one of key redox regulators in mammalian cells [16,47,48,49,50], and therefore helps protect both normal and cancer cells from oxidative stress. It appears to serve a protective function in healthy cells, and to have a tumor-promoting function when cells have transformed. It is elevated in many tumors and cancer cells, which led numerous investigators to propose this selenoenzyme as a target for cancer therapy [16,17,36,51,52,53,54,55]. However, prior to undertaking human clinical trials involving the inhibition of TXNRD1, much more understanding of the dependence of the targeted tissue on a specific antioxidant system (or systems) and the interplay between different antioxidants, antioxidant systems, and antioxidant regulatory proteins [2,3,6,8,9,15,56,57,58] are warranted. The consequences of targeting a single protein, such as TXNRD1, would most likely be the induction of NRF2, at least in some tissues [30,31,59]. NRF2 has been reported to be a transcription factor having important roles in maintaining redox balance and protecting cells from electrophilic stress (see [33,56,60] and references therein). Downregulation of NRF2 in carcinogen treated mice was reported to result in elevated tumor incidence suggesting greater susceptibility to malignancy (see [61,62] and references therein). However, in lung cancer, NRF2-deficient mice treated with the lung carcinogen, urethane, were reported to initially manifest an increase in tumor formation, whereas the corresponding NRF2-sufficient, chemically treated mice developed more tumors at later stages with a higher number of K-RAS-mutated adenocarcinomas [35]. These investigators suggested that the data revealed two roles of NRF2 during cancer development: (1) a preventive role during tumor initiation and (2) a promotion role in malignant progression.

The current study underscores the importance of elucidating the interplay between different antioxidant proteins and systems. As noted in the Introduction, the available evidence suggests that lung and liver cancers differ in how they utilize antioxidant systems to sustain malignancies [10,11,12,16,21,22,23]. Herein, we examined the two major antioxidant systems in mammals, the TXN and GSH systems, and several other known antioxidants in lung and liver tumors compared to their respective surrounding normal tissues to assess how these components changed in response to the burden of oxidative stress and enhanced growth rates. We also assessed how the tumors in these two tissues differ in utilizing these antioxidants. With regard to the TXN system, the relative amounts of TXN and PRDX1 were almost four times higher in lung tumors compared to the corresponding normal tissues, while TXN levels were approximately equal in liver tumor and normal tissues; and the levels of PRDX1 were reduced more than 50%. In addition, although lung had approximately 1.5X greater TXNRD activity in normal tissue compared to the corresponding tissue in liver, the tumor and normal tissues had similar activities in both organs. These data suggest that lung tumor and possibly the surrounding normal tissue have a greater dependence on the TXN system to protect the respective tissues from oxidative stress than liver tumor and surrounding normal tissue.

Western blot analysis revealed higher TXNRD1 levels were present in tumors compared to the respective surrounding normal tissues in both lung and liver, but, as noted above, activity assessments showed similar TXNRD activities. This observation suggested that replacement of the Sec residue with another amino acid, or truncation of TXNRD1 occurred in the malignant tissues. Unfortunately, we were not able to obtain sufficient tissue from the patients used herein to determine the amino acid replacement of the Sec residue and/or truncation of TXNRD1 to assess the influence of these parameters on TXNRD1 function, and concomitantly, on the malignancy process.

The levels of several enzymes in the GSH system and the total amount of GSH were also assessed in the various tissues. The enzymes included two selenoenzymes, GPX1 and GPX4, and several other proteins, GSR, GCLC, GSS, GLRX, GGT1, GSTA1, and GSTP1. Although the levels of several of these proteins, GPX1, GPX4, GSR, GSS, and GLRX, appeared to increase slightly in lung tumor samples compared to corresponding normal tissue, two of the proteins, GCLC and GGT1, decreased significantly in the tumors. GSTA1 was not detectable in lung tissue, however, GSTP1 increased significantly in lung tumors, indicating that GSTP1 may play a role in detoxification and maintenance of lung cancer cells. The GST family of enzymes, which uses GSH to detoxify a wide variety of molecules, are involved in kinase-mediated signaling pathways and their presence in tumors can contribute to drug resistance [42,45]. GSTP1 is widely distributed and has previously been shown to be increased in many cancer types and is the most predominantly expressed GST isozyme in the NCI-60 cancer cell line panel [45]. On the other hand, the GSH metabolic enzymes, GPX1, GSR, GCLC, GSS, GLRX, and GSTA1, were found to be downregulated in liver tumors compared to normal tissues, while GPX4 and GGT1 showed significant increases. GPX specific activities in liver were about twice those in lung, although both normal and malignant tissues had similar activities in the two organs. The total amount of GSH in lung tumor and the corresponding normal tissue was similar and about half that observed in normal liver, but normal liver tissue had three times the amount of GSH than the corresponding tumor. A large decrease in GSH levels was observed in liver within tumor tissue, while GPX activity did not increase. However, GSH levels and the GPX activity do not necessarily correlate. The activity assay determines the capacity to reduce H_2_O_2_, but within cells, activity levels are influenced by the availability of reducing equivalents for this reaction, as well as the interplay with other processes. The decrease observed in liver tumors is likely due to the decreased activity of the GSH-synthesizing enzymes, GCLC and GSS, and may also reflect an increased utilization of GSH in the tumors. GSTP1 levels were increased in two of the three liver tumor samples examined. The apparent upregulation of GSTP1 in some liver tumors may be compensatory and due, in part, to a decrease in GSTA1 in these tumors.

The changes in levels of antioxidant proteins and other antioxidants examined in lung and liver tumors compared to surrounding normal tissues are summarized in Table 3. These findings illustrate the differences in how these two tumors depend on antioxidants to drive the respective malignancy. One protein that was highly upregulated in lung and liver tumors was G6PD. G6PD is the rate-limiting enzyme of the pentose phosphate pathway, reducing NADP^+^ to NADPH, which is required for the reduction of GSH and TXN, lipid and DNA synthesis, and for certain detoxification reactions [63]. Thus, it plays a critical role in antioxidant defense, generating ribose-5-phosphate for the biosynthesis of nucleotides, and supporting other cellular functions, but its specific role in the overall malignancy process is not completely understood [64]. Recently discovered roles of G6PD in angiogenesis, cellular proliferation, and resistance to cancer therapy suggest that this protein is likely a major factor in the malignancy process [63,64]. Its highly enriched levels in the two cancers examined herein suggests that it may play a role in combating the increased oxidative stress that accompanies the respective malignancy and this observation warrants further study. SOD1 and CAT function in the reduction of superoxide to hydrogen peroxide and the conversion of hydrogen peroxide to water and oxygen, respectively [65,66]. SOD1 and CAT were not significantly altered in lung tumors, yet both were significantly reduced in liver tumors. Alterations in both SOD1 and CAT have been implicated in cancer, most likely due to their roles in modulating ROS levels [65,66]. For example, mice deficient in SOD1 have been shown to develop hepatocellular carcinomas [67].

**Table 3:** Summary of changes in levels of redox components examined in tumor and normal surrounding tissues ^1^.

Antioxidant	Lung ^2^	Liver ^2^
**TXNRD1**	↑	↑
**TXN**	↑	NS
**PRDX1**	↑	↓
**GPX1**	NS	↓
**GPX2**	ND	NS
**GPX4**	NS	↑
**GSR**	NS	↓
**GCLC**	↓	↓
**GSS**	NS	↓
**GLRX**	NS	↓
**GGT1**	↓	↑
**GSTA1**	ND	↓
**GSTP1**	↑	NS
**SOD1**	NS	↓
**CAT**	NS	↓
**G6PD**	↑	↑
**Ascorbic acid**	NS	NS
**Uric acid**	NS	↓

^1^ The observations in the table are based on quantitation of western blot analysis; ^2^ ↑ or ↓ arrows indicate significant increase (↑) or decrease (↓); NS = not significant; ND = not detected.

Several studies have shown how the interplay of different antioxidants and other redox regulators influence the malignancy process. Conrad and collaborators [18] were the first to show that malignant transformed mouse cells (mouse embryonic, fibroblast cells) required both an inhibition of TXNRD1 and the GSH system to retard the malignancy and generate a cell line more like the original, normal cells. A synergistic effect between GSH initiating cancer development and TXN promoting cancer development both *in vitro* and *in vivo* has been reported, wherein the inhibition of both systems led to cancer cell death [12]. A recent report has shown a surprising example of the interrelationship between different antioxidant proteins in mouse colon cancer cells, wherein the downregulation of either the 15kDa selenoprotein or TXNRD1 resulted in the reversal of several malignant properties to those more like normal cells; however, the double knockdown of both proteins reversed the anti-malignant properties rendering the cells with additional cancer properties [68]. Interestingly, GPX4 has been shown to have a major role in ferroptotic cancer cell death [69,70] showing that this selenoprotein also plays a role in the malignancy process. Furthermore, adequate selenium in the diet has long been correlated with cancer prevention [71], but a recent study reported that mice encoding genetically induced lung cancer and administered vitamin E and *N*-acetylcholine developed tumors much more readily and died much earlier than control mice [21]. This study also found that the two dietary antioxidants enhanced tumor growth by disrupting the association between p53 and ROS.

The present study and those discussed above strongly suggest that much more insight into the dependence of specific tissues on various antioxidants is required before undertaking human clinical trials targeting the removal of specific antioxidants or antioxidant systems. Clearly, as shown herein, there are major differences in normal and tumor tissues from lung and liver in how they utilize antioxidants to maintain a careful redox balance in normal cells and how these antioxidants are enriched and new ones induced in tumors to drive the malignancy. The observations that lung, non-small cell adenocarcinomas have highly enriched levels of TXN and PRDX1 (see Table 3), suggest that these two components provide major antioxidant support in combating the oxidative stress accompanying this specific carcinoma in lung. PRDX1 protects against oxidative stress by removing hydrogen peroxide, peroxynitrite and organic hydroperoxides, and is known to be reduced by TXN [16], supporting the proposal that non-small cell carcinoma in lung utilizes increased TXN to maintain PRDX1 in the reduced, active state. Our findings also indicate that an examination of the inhibition of TXN and/or PRDX1 as potential targets to diminish this malignancy are worthy of further consideration.

## 4. Experimental Section

### 4.1. Tissues and Other Materials

Human lung adenocarcinoma and hepatocellular carcinoma and surrounding normal tissues were purchased from ILSbio. Descriptions of these tissues and other pertinent information are given in Table 1 (lung) and Table 2 (liver).

Primary antibodies for detecting TXNRD1, GPX4, and GSR were purchased from Epitomics (Burlingame, CA, USA), for detecting GPX1, GCLC, GSS, TXN, and SOD1 from Abcam (Cambridge, MA, USA), for GPX2, GSTA1, and GSTP1 from Detroit R&D (Detroit, MI USA), for Histone H3 from Millipore (Billerica, MA, USA), for GGT1 from GeneTex (Irvine, CA, USA), for GLRX from Sigma-Aldrich (St. Louis, MO, USA), for CAT from AbFrontier (Seoul, Korea) and for G6PD and PRDX1 from Cell Signaling Technology (Danvers, MA, USA). Anti-rabbit HRP-conjugated secondary antibody was from Cell Signaling Technology. Anti-goat HRP-conjugated secondary antibody was from Sigma-Aldrich. Coomassie Blue staining solution, β-nicotinamide adenine dinucleotide phosphate reduced tetrasodium salt (NADPH), 5,5′-dithiobis (2-nitrobenzoic acid) (DNTB) and glutathione assay kit were obtained from Sigma-Aldrich and complete mini protease inhibitor cocktail from Roche (Indianapolis, IN, USA). BCA protein assay reagent, SuperSignal West Dura Extended Duration Substrate, PVDF membrane and NuPage 4%–12% Bis-Tris gels were purchased from Thermo Fisher Scientific Inc (Grand Island, NY, USA), Glutathione peroxidase activity kit was obtained from Enzo Life Science (Farmingdale, NY, USA), ascorbic acid assay kit from BioAssay System (Hayward, CA, USA) and uric acid assay kit from Thermo Fisher Scientific (Grand Island, NY, USA).

### 4.2. Tissue Preparation, Protein Concentration, and Western Blotting

Tissues were homogenized in phosphate buffered saline (PBS) with 0.5% (*v*/*v*) Triton X-100 and complete protease inhibitor cocktail, and protein amounts measured using BCA protein assay reagent. Thirty µgs of each protein sample were electrophoresed on NuPAGE Bis-Tris gels, transferred to a PVDF membrane, and then incubated initially with each primary antibody and finally with HRP-conjugated secondary antibody. Membranes were treated with SuperSignal West Dura Extended Duration Substrate and exposed to X-ray film. Following western blot analyses, membranes were stained with Coomassie Blue staining solution to evaluate protein loading and serve as a loading control. This was the preferred technique for assessing protein levels due to the large variability in expression of loading controls such as GAPDH, β-Actin and α-Tubulin [72,73,74,75,76]. We found that neither GAPDH, β-Actin, nor α-Tubulin would serve as loading controls due to their large variations in normal and malignant tissues (data not shown). The band intensities on western blots were quantified and normalized to total protein staining using ImageJ software (National Institutes of Health, Bethesda, MD, USA).

### 4.3. Enzyme Activity Assay

TXNRD activities were determined in protein extracts, which were prepared as described above, by mixing the extracts with 100 mM potassium phosphate (pH 7.0), 10 mM EDTA, 0.24 mM NADPH and 3 mM DTNB and the reduction of DTNB by TXNRD measured by absorbance at 412 nm spectrophotometrically (34). GPX activities were determined in protein extracts using a GPX activity kit (Enzo Life Science) according to the manufacturer’s instructions. The proteins were quantified with BCA protein assay reagent.

### 4.4. Determination of Total Glutathione, Ascorbic Acid, and Uric Acid Concentrations

The concentration of total glutathione (GSH + GSSG), ascorbic acid and uric acid in tumor and normal tissues were determined using the respective assay kits according to the manufacturer’s instructions. The proteins were quantified with BCA protein assay reagent.

### 4.5. Statistical Analysis

Values in all figures are presented as the standard error of the mean (SEM). Statistical analyses were performed using Student’s t-test with GraphPad Prism software (Version 5, La Jolla, CA, USA). The level of significance was set at *p* = 0.05.

## References

[1] Hanahan D., Weinberg R.A. (2011). Hallmarks of cancer: The next generation. Cell.

[2] Chandel N.S., Tuveson D.A. (2014). The promise and perils of antioxidants for cancer patients. N. Engl. J. Med..

[3] Glasauer A., Chandel N.S. (2014). Targeting antioxidants for cancer therapy. Biochem. Pharmacol..

[4] Gorrini C., Harris I.S., Mak T.W. (2013). Modulation of oxidative stress as an anticancer strategy. Nat. Rev. Drug Discov..

[5] Gupta S.C., Hevia D., Patchva S., Park B., Koh W., Aggarwal B.B. (2012). Upsides and downsides of reactive oxygen species for cancer: The roles of reactive oxygen species in tumorigenesis, prevention, and therapy. Antioxid. Redox Signal..

[6] Kardeh S., Ashkani-Esfahani S., Alizadeh A.M. (2014). Paradoxical action of reactive oxygen species in creation and therapy of cancer. Eur. J. Pharmacol..

[7] Pietraforte D., Malorni W. (2014). Focusing at the double-edged sword of redox imbalance: Signals for cell survival or for cell death?. Antioxid. Redox Signal..

[8] Sabharwal S.S., Schumacker P.T. (2014). Mitochondrial ros in cancer: Initiators, amplifiers or an achilles’ heel?. Nat. Rev. Cancer.

[9] Saeidnia S., Abdollahi M. (2013). Antioxidants: Friends or foe in prevention or treatment of cancer: The debate of the century. Toxicol. Appl. Pharmacol..

[10] Dammeyer P., Arner E.S. (2011). Human protein atlas of redox systems—What can be learnt?. Biochim. Biophys. Acta.

[11] Godoy J.R., Funke M., Ackermann W., Haunhorst P., Oesteritz S., Capani F., Elsasser H.P., Lillig C.H. (2011). Redox atlas of the mouse. Immunohistochemical detection of glutaredoxin-, peroxiredoxin-, and thioredoxin-family proteins in various tissues of the laboratory mouse. Biochim. Biophys. Acta.

[12] Harris I.S., Treloar A.E., Inoue S., Sasaki M., Gorrini C., Lee K.C., Yung K.Y., Brenner D., Knobbe-Thomsen C.B., Cox M.A. (2015). Glutathione and thioredoxin antioxidant pathways synergize to drive cancer initiation and progression. Cancer Cell.

[13] Hellfritsch J., Kirsch J., Schneider M., Fluege T., Wortmann M., Frijhoff J., Dagnell M., Fey T., Esposito I., Kolle P. (2015). Knockout of mitochondrial thioredoxin reductase stabilizes prolyl hydroxylase 2 and inhibits tumor growth and tumor-derived angiogenesis. Antioxid. Redox Signal..

[14] Kiebala M., Skalska J., Casulo C., Brookes P.S., Peterson D.R., Hilchey S.P., Dai Y., Grant S., Maggirwar S.B., Bernstein S.H. (2015). Dual targeting of the thioredoxin and glutathione antioxidant systems in malignant b cells: A novel synergistic therapeutic approach. Exp. Hematol..

[15] Kwee J.K. (2014). A paradoxical chemoresistance and tumor suppressive role of antioxidant in solid cancer cells: A strange case of Dr. Jekyll and Mr. Hyde. Biomed. Res. Int..

[16] Lu J., Holmgren A. (2014). The thioredoxin antioxidant system. Free Radic. Biol. Med..

[17] Mahmood D.F., Abderrazak A., el Hadri K., Simmet T., Rouis M. (2013). The thioredoxin system as a therapeutic target in human health and disease. Antioxid. Redox Signal..

[18] Mandal P.K., Schneider M., Kolle P., Kuhlencordt P., Forster H., Beck H., Bornkamm G.W., Conrad M. (2010). Loss of thioredoxin reductase 1 renders tumors highly susceptible to pharmacologic glutathione deprivation. Cancer Res..

[19] Peng X., Xu J., Arner E.S. (2012). Thiophosphate and selenite conversely modulate cell death induced by glutathione depletion or cisplatin: Effects related to activity and sec contents of thioredoxin reductase. Biochem. J..

[20] Traverso N., Ricciarelli R., Nitti M., Marengo B., Furfaro A.L., Pronzato M.A., Marinari U.M., Domenicotti C. (2013). Role of glutathione in cancer progression and chemoresistance. Oxid. Med. Cell. Longev..

[21] Sayin V.I., Ibrahim M.X., Larsson E., Nilsson J.A., Lindahl P., Bergo M.O. (2014). Antioxidants accelerate lung cancer progression in mice. Sci. Transl. Med..

[22] Zhao F., Yan J., Deng S., Lan L., He F., Kuang B., Zeng H. (2006). A thioredoxin reductase inhibitor induces growth inhibition and apoptosis in five cultured human carcinoma cell lines. Cancer Lett..

[23] Zhao H., Tang J., Xu J., Cao L., Jia G., Long D., Liu G., Chen X., Wang K. (2015). Selenoprotein genes exhibit differential expression patterns between hepatoma HepG2 and normal hepatocytes LO2 cell lines. Biol. Trace Elem. Res..

[24] Novoselov S.V., Calvisi D.F., Labunskyy V.M., Factor V.M., Carlson B.A., Fomenko D.E., Moustafa M.E., Hatfield D.L., Gladyshev V.N. (2005). Selenoprotein deficiency and high levels of selenium compounds can effectively inhibit hepatocarcinogenesis in transgenic mice. Oncogene.

[25] Kasaikina M.V., Turanov A.A., Avanesov A., Schweizer U., Seeher S., Bronson R.T., Novoselov S.N., Carlson B.A., Hatfield D.L., Gladyshev V.N. (2013). Contrasting roles of dietary selenium and selenoproteins in chemically induced hepatocarcinogenesis. Carcinogenesis.

[26] Brozmanova J., Manikova D., Vlckova V., Chovanec M. (2010). Selenium: A double-edged sword for defense and offence in cancer. Arch. Toxicol..

[27] Hatfield D.L., Tsuji P.A., Carlson B.A., Gladyshev V.N. (2014). Selenium and selenocysteine: Roles in cancer, health, and development. Trends Biochem. Sci..

[28] Steinbrenner H., Speckmann B., Sies H. (2013). Toward understanding success and failures in the use of selenium for cancer prevention. Antioxid. Redox Signal..

[29] Vinceti M., Crespi C.M., Malagoli C., del Giovane C., Krogh V. (2013). Friend or foe? The current epidemiologic evidence on selenium and human cancer risk. J. Environ. Sci. Health C Environ. Carcinog. Ecotoxicol. Rev..

[30] Carlson B.A., Yoo M.H., Tobe R., Mueller C., Naranjo-Suarez S., Hoffmann V.J., Gladyshev V.N., Hatfield D.L. (2012). Thioredoxin reductase 1 protects against chemically induced hepatocarcinogenesis via control of cellular redox homeostasis. Carcinogenesis.

[31] Cebula M., Schmidt E.E., Arner E.S. (2015). TrxR1 as a potent regulator of the nrf2-keap1 response system. Antioxid. Redox Signal..

[32] Dai B., Yoo S.Y., Bartholomeusz G., Graham R.A., Majidi M., Yan S., Meng J., Ji L., Coombes K., Minna J.D. (2013). Keap1-dependent synthetic lethality induced by akt and txnrd1 inhibitors in lung cancer. Cancer Res..

[33] Kansanen E., Kuosmanen S.M., Leinonen H., Levonen A.L. (2013). The Keap1-Nrf2 pathway: Mechanisms of activation and dysregulation in cancer. Redox Biol..

[34] Lau A., Villeneuve N.F., Sun Z., Wong P.K., Zhang D.D. (2008). Dual roles of Nrf2 in cancer. Pharmacol. Res..

[35] Satoh H., Moriguchi T., Takai J., Ebina M., Yamamoto M. (2013). Nrf2 prevents initiation but accelerates progression through the Kras signaling pathway during lung carcinogenesis. Cancer Res..

[36] Yoo M.H., Xu X.M., Carlson B.A., Gladyshev V.N., Hatfield D.L. (2006). Thioredoxin reductase 1 deficiency reverses tumor phenotype and tumorigenicity of lung carcinoma cells. J. Biol. Chem..

[37] Eriksson S.E., Prast-Nielsen S., Flaberg E., Szekely L., Arner E.S. (2009). High levels of thioredoxin reductase 1 modulate drug-specific cytotoxic efficacy. Free Radic. Biol. Med..

[38] Suadicani P., Hein H.O., Gyntelberg F. (2012). Serum selenium level and risk of lung cancer mortality: A 16-year follow-up of the copenhagen male study. Eur. Respir. J..

[39] Fritz H., Kennedy D., Fergusson D., Fernandes R., Cooley K., Seely A., Sagar S., Wong R., Seely D. (2011). Selenium and lung cancer: A systematic review and meta analysis. PLoS ONE.

[40] Jaworska K., Gupta S., Durda K., Muszynska M., Sukiennicki G., Jaworowska E., Grodzki T., Sulikowski M., Waloszczyk P., Wojcik J. (2013). A low selenium level is associated with lung and laryngeal cancers. PLoS ONE.

[41] Karp D.D., Lee S.J., Keller S.M., Wright G.S., Aisner S., Belinsky S.A., Johnson D.H., Johnston M.R., Goodman G., Clamon G. (2013). Randomized, double-blind, placebo-controlled, phase III chemoprevention trial of selenium supplementation in patients with resected stage I non-small-cell lung cancer: ECOG 5597. J. Clin. Oncol..

[42] Huang S.X., Wu F.X., Luo M., Ma L., Gao K.F., Li J., Wu W.J., Huang S., Yang Q., Liu K. (2013). The glutathione s-transferase p1 341c>t polymorphism and cancer risk: A meta-analysis of 28 case-control studies. PLoS ONE.

[43] Lu S.C. (1999). Regulation of hepatic glutathione synthesis: Current concepts and controversies. FASEB J..

[44] Schnekenburger M., Karius T., Diederich M. (2014). Regulation of epigenetic traits of the glutathione s-transferase p1 gene: From detoxification toward cancer prevention and diagnosis. Front. Pharmacol..

[45] Singh S. (2015). Cytoprotective and regulatory functions of glutathione s-transferases in cancer cell proliferation and cell death. Cancer Chemother. Pharmacol..

[46] Balendiran G.K., Dabur R., Fraser D. (2004). The role of glutathione in cancer. Cell Biochem. Funct..

[47] Arner E.S., Holmgren A. (2000). Physiological functions of thioredoxin and thioredoxin reductase. Eur. J. Biochem..

[48] Arner E.S.J. (2009). Focus on mammalian thioredoxin reductases—Important selenoproteins with versatile functions. Biochim. Biophys. Acta Gen. Subj..

[49] Rundlof A.K., Arner E.S. (2004). Regulation of the mammalian selenoprotein thioredoxin reductase 1 in relation to cellular phenotype, growth, and signaling events. Antioxid. Redox Signal..

[50] Rundlof A.K., Janard M., Miranda-Vizuete A., Arner E.S. (2004). Evidence for intriguingly complex transcription of human thioredoxin reductase 1. Free Radic. Biol. Med..

[51] Arner E.S.J., Holmgren A. (2006). The thioredoxin system in cancer. Semin. Cancer Biol..

[52] Biaglow J.E., Miller R.A. (2005). The thioredoxin reductase/thioredoxin system: Novel redox targets for cancer therapy. Cancer Biol. Ther..

[53] Carlson B.A., Brigelius-Flohe R., Sies H. (2015). Dual functions of selenoproteins in cancer: Thioredoxin reductase. Diversity of Selenium Functions in Health and Disease.

[54] Gromer S., Urig S., Becker K. (2004). The thioredoxin system—From science to clinic. Med. Res. Rev..

[55] Nguyen P., Awwad R.T., Smart D.D., Spitz D.R., Gius D. (2006). Thioredoxin reductase as a novel molecular target for cancer therapy. Cancer Lett..

[56] Itoh K., Mimura J., Yamamoto M. (2010). Discovery of the negative regulator of Nrf2, Keap1: A historical overview. Antioxid. Redox Signal..

[57] Kensler T.W., Wakabayashi N. (2010). Nrf2: Friend or foe for chemoprevention?. Carcinogenesis.

[58] Moon E.J., Giaccia A. (2015). Dual roles of Nrf2 in tumor prevention and progression: Possible implications in cancer treatment. Free Radic. Biol. Med..

[59] Suvorova E.S., Lucas O., Weisend C.M., Rollins M.F., Merrill G.F., Capecchi M.R., Schmidt E.E. (2009). Cytoprotective Nrf2 pathway is induced in chronically txnrd 1-deficient hepatocytes. PLoS ONE.

[60] Murakami S., Motohashi H. (2015). Roles of Nrf2 in cell proliferation and differentiation. Free Radic. Biol. Med..

[61] Osburn W.O., Karim B., Dolan P.M., Liu G., Yamamoto M., Huso D.L., Kensler T.W. (2007). Increased colonic inflammatory injury and formation of aberrant crypt foci in Nrf2-deficient mice upon dextran sulfate treatment. Int. J. Cancer.

[62] Xu C., Huang M.T., Shen G., Yuan X., Lin W., Khor T.O., Conney A.H., Kong A.N. (2006). Inhibition of 7,12-dimethylbenz(a)anthracene-induced skin tumorigenesis in c57bl/6 mice by sulforaphane is mediated by nuclear factor e2-related factor 2. Cancer Res..

[63] Zhang C., Zhang Z., Zhu Y., Qin S. (2014). Glucose-6-phosphate dehydrogenase: A biomarker and potential therapeutic target for cancer. Anticancer Agents Med. Chem..

[64] Jiang P., Du W., Wu M. (2014). Regulation of the pentose phosphate pathway in cancer. Protein Cell.

[65] Glorieux C., Zamocky M., Sandoval J.M., Verrax J., Calderon P.B. (2015). Regulation of catalase expression in healthy and cancerous cells. Free Radic. Biol. Med..

[66] Papa L., Manfredi G., Germain D. (2014). Sod1, an unexpected novel target for cancer therapy. Genes Cancer.

[67] Elchuri S., Oberley T.D., Qi W., Eisenstein R.S., Jackson Roberts L., van Remmen H., Epstein C.J., Huang T.T. (2005). Cuznsod deficiency leads to persistent and widespread oxidative damage and hepatocarcinogenesis later in life. Oncogene.

[68] Tsuji P.A., Carlson B.A., Yoo M.H., Naranjo-Suarez S., Xu X.M., He Y., Asaki E., Seifried H.E., Reinhold W.C., Davis C.D. (2015). The 15kda selenoprotein and thioredoxin reductase 1 promote colon cancer by different pathways. PLoS ONE.

[69] Friedmann Angeli J.P., Schneider M., Proneth B., Tyurina Y.Y., Tyurin V.A., Hammond V.J., Herbach N., Aichler M., Walch A., Eggenhofer E. (2014). Inactivation of the ferroptosis regulator Gpx4 triggers acute renal failure in mice. Nat. Cell Biol..

[70] Yang W.S., SriRamaratnam R., Welsch M.E., Shimada K., Skouta R., Viswanathan V.S., Cheah J.H., Clemons P.A., Shamji A.F., Clish C.B. (2014). Regulation of ferroptotic cancer cell death by Gpx4. Cell.

[71] Jackson M.I., Combs G.F.J., Hatfield D.L., Berry M.J., Gladyshev V.N. (2012). Selenium as a cancer preventive agent. Selenium: Its Molecular Biology and Role in Human Health.

[72] Aldridge G.M., Podrebarac D.M., Greenough W.T., Weiler I.J. (2008). The use of total protein stains as loading controls: An alternative to high-abundance single-protein controls in semi-quantitative immunoblotting. J. Neurosci. Methods.

[73] Collins M.A., An J., Peller D., Bowser R. (2015). Total protein is an effective loading control for cerebrospinal fluid western blots. J. Neurosci. Methods.

[74] Eaton S.L., Roche S.L., Llavero Hurtado M., Oldknow K.J., Farquharson C., Gillingwater T.H., Wishart T.M. (2013). Total protein analysis as a reliable loading control for quantitative fluorescent western blotting. PLoS ONE.

[75] Klein D., Kern R.M., Sokol R.Z. (1995). A method for quantification and correction of proteins after transfer to immobilization membranes. Biochem. Mol. Biol. Int..

[76] Welinder C., Ekblad L. (2011). Coomassie staining as loading control in western blot analysis. J. Proteome Res..

